# Our Experience in Diagnosing and Treating Breast Implant-Associated Anaplastic Large Cell Lymphoma (BIA-ALCL)

**DOI:** 10.3390/jcm13020366

**Published:** 2024-01-09

**Authors:** Gon Shoham, Oriana Haran, Roei Singolda, Ehab Madah, Ada Magen, Orit Golan, Tehillah Menes, Ehud Arad, Yoav Barnea

**Affiliations:** 1 Department of Plastic and Reconstructive Surgery, Tel Aviv Sourasky Medical Center, Affiliated with the Faculty of Medicine, Tel Aviv University, Tel Aviv 6997801, Israel; 2 Breast Health Center, Tel Aviv Sourasky Medical Center, Affiliated with the Faculty of Medicine, Tel Aviv University, Tel Aviv 6423906, Israel; 3 Breast Imaging Center, Tel Aviv Sourasky Medical Center, Affiliated with the Faculty of Medicine, Tel Aviv University, Tel Aviv 6423906, Israel; 4 Meirav Breast Center, Sheba Medical Center, Affiliated with the Faculty of Medicine, Tel Aviv University, Tel Hashomer 5262000, Israel

**Keywords:** breast, BIA-ALCL, breast implants, lymphoma, breast augmentation

## Abstract

Breast implant-associated anaplastic large cell lymphoma (BIA-ALCL) is an uncommon T-cell lymphoma detected in association with textured implants. It presents as a fluid accumulation around the implant, usually years after the implantation. We present our experience in diagnosing and treating four patients with BIA-ALCL, each widely differing from the other. Data on patients’ surgical history, relevant medical information, and findings on pathological slides were retrieved from their medical charts and retrospectively reviewed. Each of the four patients was diagnosed with BIA-ALCL, one after breast augmentation, one after breast reconstruction with an implant, one after breast reconstruction with a latissimus dorsi flap and implant, and the fourth after the removal of breast implants. The cases were presented to a multidisciplinary team and subsequently underwent surgery. All four are currently free of tumors, as established by a negative follow-up via positron emission tomography-computed tomography. Although the incidence of BIA-ALCL is rare, these cases emphasize the need to rule out the diagnosis of BIA-ALCL in patients with textured implants or a history of implanted textured devices who present with symptoms such as late seroma or peri-implant mass. This pathology is typically indolent and slow-growing and heightened awareness for an early diagnosis could lead to quicker intervention and enhanced patient management.

## 1. Introduction

First reported in 1997, breast implant-associated anaplastic large cell lymphoma (BIA-ALCL) has emerged as a noteworthy medical phenomenon, primarily manifesting as an accumulation of seroma fluid situated between the breast implant and the surrounding fibrous capsule [1]. The scale of this condition gained significant attention, with a cumulative global report of 1130 cases according to the 2022 Food and Drug Administration (FDA) data. Intriguingly, BIA-ALCL exhibits a distinctive association with women who have textured-surface implants, or a history thereof, predominantly filled with silicone; however, more than 25% of cases involve saline-filled implants, adding complexity to its understanding. The median time from the implantation to diagnosis averages 8 years, though instances have been documented with durations spanning from 1 to an astonishing 34 years [2].

Divergent views on the lifetime risk of developing BIA-ALCL have surfaced in recent studies, with Cordeiro et al.’s retrospective analysis of 3546 women indicating a heightened risk, where 1 in 355 women developed BIA-ALCL [3]. Contrarily, a recently published study by Kolasiński et al. documented 5 cases among 1501 cosmetic breast surgery recipients, resulting in a prevalence rate of 1 in 300 patients [4]. These findings, together with data from implant registries demonstrating that macrotextured Allergan Biocell® (Allergan, Abbvie Inc., North Chicago, IL, USA) implants had a disproportionately higher risk for BIA-ALCL, led the FDA to recall these devices in 2019 [5]. This drove the company to voluntarily remove these products from the global market. The most common clinical presentation of BIA-ALCL was delayed periprosthetic effusion (80%), followed by breast swelling and pain (23%), capsule contracture (13%), and a peri-implant mass or lump (11%) [2,6]. The patients’ symptoms usually occurred at least one-year post-implantation, with an average of 10 years [7].

In the context of this intriguing medical landscape, our study contributes a unique perspective by detailing the diagnostic and treatment experiences of four patients with BIA-ALCL, each presenting distinct clinical features. While the incidence of BIA-ALCL is rare, the varied presentations underscore the imperative need to consider and rule out this diagnosis in patients with textured implants or a history of such devices or those who exhibit symptoms like late seroma, breast swelling, capsular contracture, or peri-implant mass. Our exploration of these diverse cases adds a nuanced layer to an evolving understanding of BIA-ALCL, emphasizing the importance of vigilance and diagnostic precision in managing this rare but impactful medical condition.

## 2. Materials and Methods

In our study, we meticulously enrolled four patients diagnosed with confirmed breast implant-associated anaplastic large cell lymphoma (BIA-ALCL) who received treatment at our medical center during the period spanning from 2020 to 2023. The age range of these individuals at the time of diagnosis varied from 48 to 74 years, reflecting the diverse demographic profile of the affected population.

Within our department, a standardized protocol is followed for all patients suspected of having BIA-ALCL. This entails routine breast imaging, employing either ultrasound (US) or magnetic resonance imaging (MRI). In the presence of a detected fluid, a fine needle aspiration (FNA) procedure is conducted, aiming to collect a minimum of 50 cc for an exhaustive evaluation. This includes an assessment of cell morphology through cytology, an examination of CD30 and anaplastic lymphoma kinase (ALK) expression via immunohistochemistry, and an evaluation, quantification, and characterization of T cells and additional biomarkers through flow cytometry [8,9].

All the confirmed BIA-ALCL cases undergo comprehensive discussion in a multidisciplinary meeting, bringing together hemato-oncologists, breast surgeons, breast imaging specialists, pathologists, and plastic surgeons. As part of the diagnostic process, preoperative positron emission tomography-computed tomography (PET-CT) scans are conducted to facilitate oncologic staging and to rule out the presence of associated capsular masses, lymph node involvement, and distant metastasis [10].

The treatment approach involves “en-bloc capsulectomy”, a procedure that includes the resection of the implant along with the surrounding fibrous capsule and any associated capsule mass as a whole. This oncological resection is executed in collaboration with a breast surgeon. Surgically excised tissue and aspirated fluid are subsequently sent for further analysis to determine the tumor margins and disease stage [11].

Post-surgery, patients undergo vigilant surveillance, encompassing both surgical and hemato-oncological follow-ups. All pertinent data are meticulously stored in electronic medical records, ensuring accessibility for retrospective gathering and analysis. This comprehensive approach not only underscores the importance of early detection and treatment but also emphasizes the significance of a multidisciplinary strategy in managing BIA-ALCL for the best possible patient outcomes.

## 3. Results

### 3.1. Case 1: BIA-ALCL after Breast Augmentation

This 64-year-old woman with a background of dyslipidemia who was treated with alirocumab, aspirin, and gabapentin was a non-smoker whose maternal aunt was diagnosed with breast cancer. The patient’s surgical history included a cosmetic bilateral breast augmentation at age 49 years. The size, surface, and manufacturer of the implants were unknown. They had been placed in a sub-glandular plane. One year following that surgery, she experienced right capsular contracture and underwent bilateral implant exchange, switching to Biocell® style 510 MX 490 cc implants (Allergan, Abbvie Inc., North Chicago, IL, USA), and position change to the sub-muscular plane.

**Figure 1:**
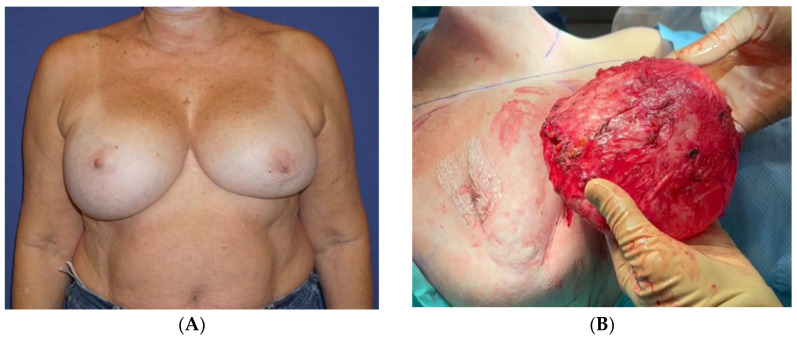

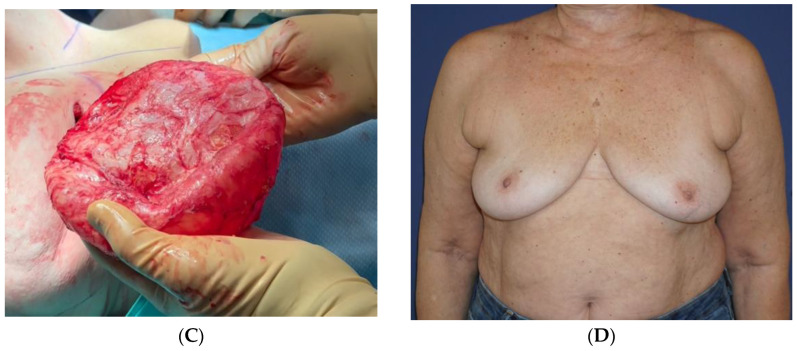
A 64-year-old woman that underwent breast augmentation using Allergan Biocell® im-plants 15 years ago. She developed a late seroma on the right breast (**A**) that was diagnosed as BIA-ALCL. She underwent bilateral implant removal with a right en-bloc capsulectomy (**B**,**C**). The patient 6 months after the surgery (**D**).

Thirteen years following the second surgery, she experienced headaches, dizziness, fatigue, and swelling in her right breast (Figure 1A). She underwent an MRI scan, which detected an intracapsular rupture in the left implant and a significant amount of fluid surrounding the intact right implant. There was no sign of enlarged axillary lymph nodes. An ultrasound (US) scan and an FNA were performed on the right breast. The aspirated fluid showed enlarged cells with atypical morphology and positive CD30, CD43, and CD4 markers, consistent with BIA-ALCL. A PET-CT scan revealed a nonspecific uptake in the surrounding areas of the implants, most probably related to a reactive response, with no axillary or distant uptake. Following a multidisciplinary consultation, the patient was advised to remove both implants and capsules, to which she agreed. The surgery was conducted by a plastic surgeon in collaboration with a breast surgeon and included a bilateral capsulectomy and implant removal with an intact, en-bloc capsulectomy on the right side (Video S1, Figure 1B,C).

The pathology report concluded that the right breast’s inner capsule demonstrated fibrosis and lymphocytic infiltration. The biopsied cells were positive for CD3 and negative for CD20, CD30, and ALK. CD68 was positive in histiocytes. The cells that were detached from the capsule and did not infiltrate it were positive for CD30. The fluid cytology of the capsule clearly showed cells with an atypical morphology, and it was positive for CD30 immunoreaction. Therefore, the diagnosis of BIA-ALCL was confined to the effusion, without evidence of infiltration, and staged as tumor-node metastasis (TNM) 1A. The left breast showed no signs of lymphoma. The patient’s subsequent course of recovery was uneventful (Figure 1D). The PET-CT performed 6 months after the surgery was normal, with no pathological findings.

### 3.2. Case 2: BIA-ALCL after Breast Implant Reconstruction

This 57-year-old otherwise healthy woman was a non-smoker who did not regularly consume medications. Her mother was diagnosed with breast cancer at age 36 years, and she was a BRCA1 mutation carrier. The patient underwent a prophylactic risk-reducing salpingo-oophorectomy and a bilateral skin-sparing mastectomy followed by immediate reconstruction with Biocell® implants (Allergan, Abbvie Inc., North Chicago, IL, USA) in a dual plane fashion using a Seri® surgical scaffold (Sofregen Medical Inc., Framingham, MA, USA). Shortly afterward, she developed bilateral wound dehiscence and an infection in the left breast. She underwent the debridement and irrigation of the wounds, and both implants were exchanged with the Biocell® expandable implants 495-520 cc style 150 (Allergan, Abbvie Inc., North Chicago, IL, USA). She healed uneventfully.

**Figure 2:**
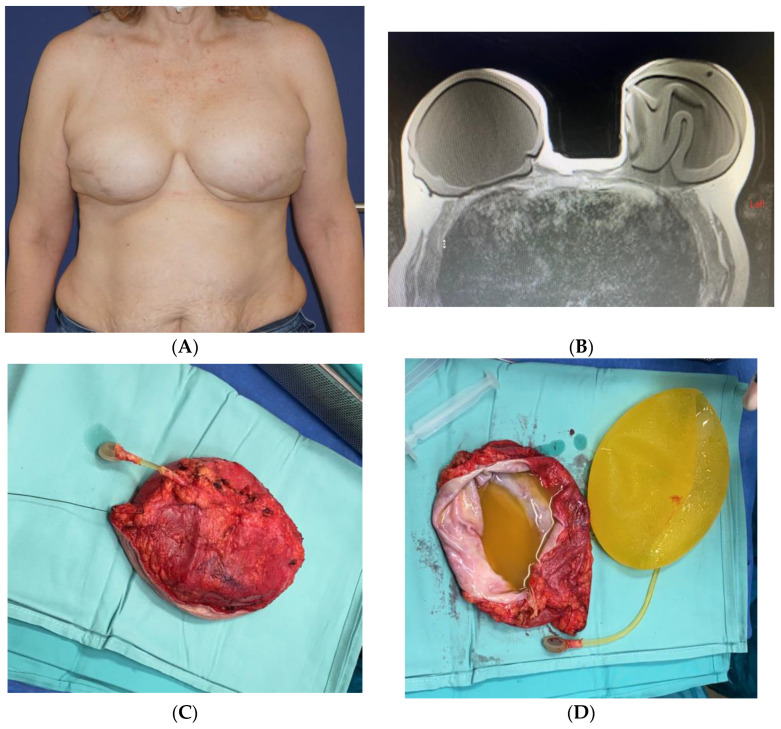
A 57-year-old BRCA carrier that underwent a bilateral risk-reducing mastectomy and immediate reconstruction with Allergan Biocell® implants and ADM. She developed a late seroma on the left breast 7 years after surgery (**A**) that was diagnosed as BIA-ALCL. On the MRI, the left breast demonstrated the collapse of the implant with abundant fluid surrounding it (**B**). She underwent bilateral implant removal with a left en-bloc capsulectomy (**C**). The fluid surrounding the implant was taken for cytology testing, and the capsule was taken for histology (**D**).

Seven years after the initial surgery, the patient reported flu-like symptoms, followed by an enlargement of the left breast, resulting in marked asymmetry (Figure 2A). An US scan revealed that the implant was surrounded by fluid, and an FNA extracted 80 mL of cloudy fluid. The cytology report showed an atypical lymphoid population highly suspicious of BIA-ALCL. A PET-CT scan showed weak absorption in the area surrounding the left implant, with a focused area of hyperabsorption, in addition to the weak absorption of a lymph node in the left axilla. An MRI scan demonstrated a large amount of fluid surrounding the left implant, with no suspicious enhancement (Figure 2B). Both implants appeared to be intact, and there were no suspicious axillary lymph nodes.

Following a multidisciplinary consultation, the patient was referred to surgical treatment. Surgery included a left-hand en-bloc capsulectomy (intact implant and capsule), a left axilla sentinel lymph node biopsy in collaboration with a breast surgeon, and the right-hand removal of the implant and capsule (Figure 2C,D). The excess breast skin was resected bilaterally. The pathology report indicated no sign of lymphoma in the left capsule or axillary lymph node; however, the fluid from the area surrounding the left implant showed large atypical cells that were positive for CD30, negative for ALK, and negative for CD2, CD3, CD4, CD7, CD8, CD20, CD56, and TIA-1. Many of the large cells were positive for Ki-67. An additional pathology specialist was consulted and concluded that the findings were highly suggestive of BIA-ALCL confined to the effusion without evidence of infiltration (BIA-ALCL TNM stage 1A).

The patient’s course of recovery was uneventful. A PET-CT was performed one year after surgery, and it did not reveal any sign of lymphoma. The standard follow-up breast US also failed to reveal any suspicious findings.

### 3.3. Case 3: BIA-ALCL after Implant Removal

This 48-year-old woman, who was generally in good health, did not regularly take medications, smoked occasionally, and had no family history of breast cancer. In terms of her surgical history, she underwent a cosmetic bilateral mastopexy augmentation at the age of 28. During this procedure, she received 380 cc textured-surface implants from an unspecified manufacturer, placed in the sub-glandular plane. Dissatisfied with the implant size, she opted for an implant exchange one year later, downsizing to a 305 cc implant (textured, brand not disclosed) in the same sub-glandular plane, along with a re-mastopexy. Three years thereafter, she underwent another implant exchange, this time receiving Allergan Biocell®-textured round TSM 210 cc implants (Allergan, Abbvie Inc., North Chicago, IL, USA), along with another re-mastopexy.

**Figure 3:**
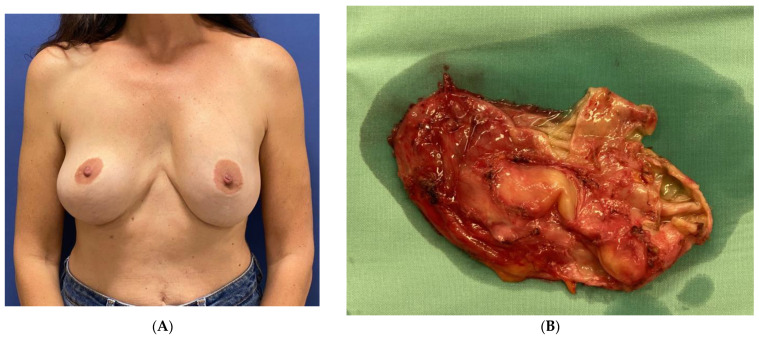

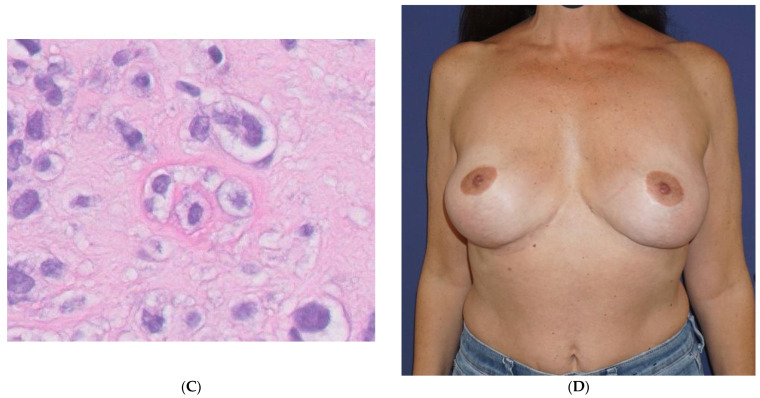
A 48-year-old woman that underwent augmentation mastopexy using Allergan Biocell® implants. She developed a left breast late seroma 6 years after the implant removal (**A**). She underwent a left en-bloc excision of the encapsulated seroma (**B**). The histopathology from the capsule showing sheets of large atypical cells on the inside surface of the capsule compatible with BIA-ALCL (H&E ×400) (**C**). The patient 15 months after surgery (**D**) [12].

Ten years after her last surgery, she exhibited pain, swelling, and discomfort in her left breast without any indications of infection or a previous history of local trauma. She underwent US imaging of the left breast and an FNA of 50 cc for serous fluid. No specific staining was performed at that time, and the condition was diagnosed as benign. By the patient’s decision, both implants were removed together with the surrounding inner capsule, and a left breast outer capsulectomy was performed. Healing was uneventful. No pathological review was possible due to a lack of specimens from that surgery. After six years, the patient reported a 3-month duration of painful swelling in the upper left breast without any recent incidents of trauma, strenuous physical activity, or infection (Figure 3A). The US scan identified a 9.5 cm sub-glandular fluid collection and 100 cc of clear serous fluid was aspirated. The cytology report indicated the existence of amorphous acellular material with no signs of malignancy. No staining for CD30 immunohistochemistry was performed since the specimen was negative for atypical lymphocytes. The seroma recurred 3 weeks after the drainage procedure. An intact seroma cavity was then removed surgically with an en-bloc capsulectomy containing an estimated 200 cc of serous fluid (Figure 3B). The inner surface of the capsule revealed a smooth luminal surface with fibrous tissue and fibrin cords. There were no signs of free silicone or silicone granulomas.

The fluid underwent cytology and bacteriology testing, while the capsule underwent a histological evaluation. The cytology report revealed the presence of large atypical cells positive for CD30 and CD3 and negative for ALK, consistent with the diagnosis of BIA-ALCL. The bacterial cultures from the serous fluid returned negative. The pathology report on the capsule described a fibrous tissue with a fibrinous covering, small-size lymphocytes, and clusters of large, atypical, invasive cells in the fibrous tissue, indicative of BIA-ALCL TNM stage 1B. These cells exhibited positivity for CD30 and CD3 and negativity for ALK (Figure 3C). The cytology report of the seroma of the left breast, taken back in 2014, was subsequently revised to describe the presence of large-cell morphology lymphocytes compatible with BIA-ALCL. The patient’s course of recovery was uneventful (Figure 3D). One month following the last surgery, a PET-CT was conducted, uncovering a nonspecific uptake in the left breast without evidence of regional or distant metastasis.

### 3.4. Case 4: BIA-ALCL after a Latissimus Dorsi Flap and Implant Reconstruction

This 74-year-old otherwise healthy woman had a history of smoking up to the age of 40 and occasionally consumed proton pump inhibitors. She underwent a left-side lumpectomy due to a breast malignancy 17 years ago, followed by local radiation. A year later, tumor recurrence was diagnosed, resulting in a skin-sparing mastectomy, an immediate reconstruction with a Latissimus Dorsi myocutaneous flap, an Allergan Biocell®-textured round ST-120 280 cc implant (Allergan, Abbvie Inc., North Chicago, IL, USA), and a contralateral mammoplasty.

**Figure 4:**
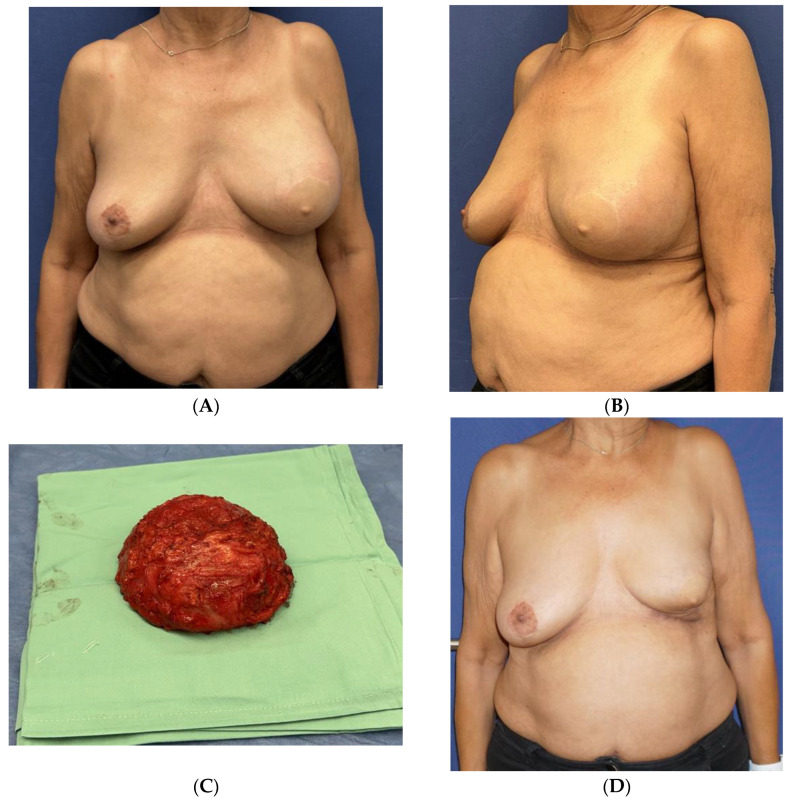
A 74-year-old underwent a skin-sparing mastectomy and immediate reconstruction with a Latissimus Dorsi myocutaneous flap and an Allergan Biocell® implant. She developed a late seroma on the left breast 16 years after the surgery (**A**,**B**) that was diagnosed as BIA-ALCL. She underwent left implant removal with an en-bloc capsulectomy (**C**). She continued a multidisciplinary follow-up (**D**).

Following complaints of swelling and hardening in her left breast (Figure 4A,B), she underwent US and mammography exams, which demonstrated an intact implant and abundant extra-capsular fluid. The fluid was aspirated under an US and sent for cytology analysis and markers. The report indicated the presence of anaplastic cells, positive to CD30, CD3, and negative to ALK, compatible with the diagnosis of BIA-ALCL. A PET-CT scan demonstrated a limited uptake in the area surrounding the implant, with no axillary or distant uptake. A left breast en-block capsulectomy was performed (Figure 4C, Video S2) with the diagnosis of BIA-ALCL; the implant capsule was focally infiltrated by atypical large cells positive for CD30 with a high Ki-67 labeling index; CD2 and CD4 were weakly positive; and stains for CD3, CD5, CD8, CD7, CD20, and CK were negative, as well as ALK.

She continued a multidisciplinary follow-up (Figure 4D) with normal US and PET-CT exams 6 months after the surgery.

## 4. Discussion

Breast implants have been on the market for over 50 years presenting many benefits, both in the reconstructive and aesthetic fields. Questions have been raised regarding the long-term safety of breast implants, including their associations with BIA-ALCL [13]. These concerns led to the 2019 FDA’s recall of Allergen Biocell® devices (Allergan, Abbvie Inc., North Chicago, IL, USA). Although the criteria for a BIA-ALCL diagnosis are well established, the extent of the risk remains unclear and varies extensively among publications [3]. Moreover, even though more than two decades have passed since the first report of BIA-ALCL, the pathophysiology and the molecular mechanisms responsible for aberrant T-cell clonal expansion remain poorly understood [14].

The treatment of BIA-ALCL with the complete intact surgical excision of the capsule is associated with exceptionally long-term disease-free survival. When diagnosed early and when the disease is localized to the capsule or the peri-prosthetic fluid, treatment can be confined to an en-bloc resection alone. However, in rare cases when there are signs of infiltration beyond the capsule, the recurrence is 2.6-fold higher, and it increases to 2.7-fold higher when more than one local or regional lymph node is involved [11]. An early diagnosis and surgical treatment are even more imperative since there are no agreed-upon guidelines for adjuvant treatment [8].

We present four highly diverse cases of BIA-ALCL diagnosed in June 2022, April 2020, July 2020, and April 2023, respectively. The first patient underwent cosmetic breast augmentation and was diagnosed after presenting with unilateral breast swelling. The second patient underwent a bilateral prophylactic mastectomy with immediate implant reconstruction and later developed flu-like symptoms and swelling of the left breast that was diagnosed as BIA-ALCL. The third patient presented with unilateral breast pain and swelling symptoms after mastopexy–augmentation and was misdiagnosed as having a benign seroma (considered initially as a classic case of late seroma before the BIA-ALCL era), eventually undergoing implant removal and an incomplete capsule resection. Six years after implant removal, with the return of symptoms, the patient underwent an en-bloc resection of both the seroma cavity and the capsule and was diagnosed with BIA-ALCL. The fourth patient underwent a mastectomy with an immediate Latissimus Dorsi flap and implant reconstruction following breast cancer recurrence; she presented with swelling and hardening in her left breast.

The reasons for implant-based surgery varied among these patients. However, an interesting finding that united them was that all four had Biocell® devices (Allergan, Abbvie Inc., North Chicago, IL, USA), and three underwent multiple surgeries with implant exchange. The current proposed etiology pathway for BIA-ALCL is bacterial contamination, which, via bacterial adhesion, leads to a higher rate of lymphocyte activation [15]. We, therefore, hypothesize that multiple surgeries may increase the possibility of implant contamination. On the other hand, the initial findings from biofilm studies could not be replicated, and subsequent studies have demonstrated that there is no distinct patient-specific microbiome [16]. This has led to the role of biofilm in BIA-ALCL being downplayed in recent years; moreover, regarding multiple surgeries, current evidence suggests quite the opposite, as implant replacements have been found to have a protective role against BIA-ALCL, reducing incidence rate and risk, while increasing event-free survival [17].

The intriguing nature of BIA-ALCL as a research question arises from the diversity of theories and the intricacies of its pathophysiology.

BIA-ALCL has many types of clinical presentations, including a diverse patient history of textured implants and the varied timing of diagnosis, from several years following breast implantation to several years following breast implant removal [12,18]. Several diagnostic workflows have been suggested, and different treatment protocols are recommended for symptomatic and asymptomatic patients with textured implants [8,19]. Asymptomatic patients with textured implants do not need to remove their implants and can follow regular implant imaging surveillance for rupture or fluid collection [12,20]. Patients should be alert to detect any change or symptom related to the implant, such as late seroma, breast mass, implant hardening, etc. Patients without symptoms who are considering implant removal should be informed about the potential risks associated with total capsulectomy. It is worth noting that a recent review indicated that the risk of reoperation for prophylactic capsulectomy and the removal of breast implants in individuals with high-risk textured devices is not greater than the risk of reoperation for therapeutic capsulectomy following a breast implant removal or an exchange-prompted by implant complications [20].

Symptomatic patients, especially those with late seroma, should undergo a thorough preoperative investigation of the fluid. Moreover, there should be a high level of suspicion for patients with Biocell®-textured devices or a history of Biocell®-textured devices (Allergan, Abbvie Inc., North Chicago, IL, USA). The pathologist should receive all the relevant clinical data and be mindful to rule out BIA-ALCL. A total capsulectomy, either intact or separated from the implant, is reasonable for patients with negative findings for BIA-ALCL [20], but capsules should be sent for histological evaluation. Symptomatic patients with a BIA-ALCL diagnosis should be discussed within a multidisciplinary team to further plan diagnosis and treatment. Good communication among specialists is essential to expedite diagnosis, with the plastic surgeon serving as the case manager and coordinator.

The recommended surgical treatment includes an oncological en-bloc resection, emphasizing the need to completely remove the capsule and additionally involve tissue as needed [21]. Recent treatment algorithms have included recommendations regarding reconstruction as well. Lamaris et al. [22] advised that all patients should be candidates for reconstruction following a resection, including the use of smooth implants, and the severity of the disease should determine the timing of the reconstruction. Finally, given the uncommonness of this pathology, it is vital that all BIA-ALCL cases be registered in the national implant registry. While Israel currently does not have a national registry, there is a position paper written by the society regarding late seroma and BIA-ALCL diagnosis and treatment. These recommendations parallel the American Society of Plastic Surgery’s (ASPS) recommendations [23]. For each case of BIA-ALCL, there is an ad-hoc multidisciplinary committee discussing the treatment options and follow-up. Moreover, surgeons are encouraged to report to the FDA as a Medical Device Report (MDR), according to the widely accepted guidelines [24].

The cornerstone for achieving early detection and optimal treatment outcomes in BIA-ALCL lies in fostering education and awareness among both patients and healthcare professionals. By enhancing knowledge about the condition, individuals can recognize the potential symptoms promptly, seek timely medical attention, and actively participate in their healthcare journey. Simultaneously, healthcare providers equipped with a heightened awareness of BIA-ALCL can facilitate swift diagnoses and implement appropriate treatment strategies, thus significantly improving the prospects of a full and successful recovery for affected patients. Hence, investing in educational initiatives and fostering awareness campaigns emerges as a pivotal strategy in the comprehensive approach to managing and mitigating the impact of BIA-ALCL.

## 5. Conclusions

In this comprehensive exploration of our clinical encounters, we aim to contribute to the ongoing discourse surrounding BIA-ALCL, shedding light on the nuances that enhance physician’s awareness and diagnostic acumen. BIA-ALCL, characterized by its typically indolent and slow-growing nature, underscores the paramount importance of vigilance in recognizing subtle yet potentially significant symptoms.

Our collective experience in diagnosing and managing BIA-ALCL serves as a crucial reminder to the medical community about the subtleties inherent in this condition. The prognosis, reassuringly, leans heavily towards positive long-term survival outcomes, further underscoring the significance of early detection and intervention.

Symptoms indicative of BIA-ALCL, such as asymmetrical breast pain and swelling, implant hardening, or the presence of a peri-implant mass or lump, beckon for heightened clinical suspicion. This is particularly pertinent in patients boasting medical histories that involve the use of textured implants. Recognizing the potential correlation between these symptoms and BIA-ALCL becomes pivotal in hastening the diagnostic process and facilitating prompt and effective treatment strategies.

By revisiting our encounters with BIA-ALCL cases, we aim not only to share clinical insights but also to foster a renewed sense of awareness among physicians. This renewed awareness, in turn, can catalyze proactive diagnostic efforts and timely interventions and ultimately contribute to the overarching goal of optimizing patient outcomes in the realm of breast health. As we navigate the evolving landscape of medical knowledge, our commitment to sharing experiences serves as a linchpin in fortifying the medical community’s collective understanding of BIA-ALCL.

## Data Availability

The raw data supporting the conclusions of this article will be made available by the authors on request.

## Supplementary Materials

The following supporting information can be downloaded at: https://www.mdpi.com/article/10.3390/jcm13020366/s1, Video S1 (case 1): A 64-year-old woman that underwent breast augmentation using Allergan Biocell® implants 15 years ago. She developed a late seroma on the right breast that was diagnosed as BIA-ALCL. She underwent bilateral implant removal with a right en-bloc capsulectomy. Video S2 (case 2): A 74-year-old underwent a skin-sparing mastectomy and immediate reconstruction with a Latis-simus Dorsi myocutaneous flap and an Allergan Biocell® implant. She developed a late seroma on the left breast 16 years after the surgery that was diagnosed as BIA-ALCL. She underwent left implant removal with an en-bloc capsulectomy, the capsule was opened for inspection during the surgery.
